# Influence of a portable X-ray device in the diagnosis of proximal caries lesions

**DOI:** 10.1590/1807-3107bor-2025.vol39.028

**Published:** 2025-05-23

**Authors:** Débora Costa RUIZ, Rocharles Cavalcante FONTENELE, Amanda FARIAS-GOMES, Hugo GAÊTA-ARAUJO, Matheus Lima OLIVEIRA, Deborah Queiroz FREITAS, Francisco HAITER-NETO

**Affiliations:** (a) Universidade Estadual de Campinas – Unicamp, Piracicaba Detal School, Department of Oral Diagnosis, Piracicaba, SP, Brazil.; (b) OMFS IMPATH Research Group, School of Medicine, Department of Imaging and Pathology, Leuven, Belgium; (c) Universidade de São Paulo – USP, Ribeirão Preto School of Dentistry, Department of Stomatology, Public Health, and Forensic Dentistry, Ribeirão Preto, SP, Brazil.

**Keywords:** X-Rays, Radiography, Dental, Digital, Diagnostic Imaging, Dental Caries

## Abstract

This study aimed to evaluate the influence of a portable X-ray device on the diagnosis of proximal caries lesions. For that, radiographs of 40 human teeth with white spots or color changes in enamel and/or dentin were acquired using the Eagle X-ray portable device (Alliage, São Paulo, Brazil) set at 2.5 mA, 60 kVp and an exposure time of 0.5 s (1.25 mAs). Then, new radiographs of the teeth were acquired using the Focus X-ray wall-mounted device (Instrumentarium, Tuusula, Finland) set at 7 mA, 70 kVp, and exposure time of 0.16 s (1.12 mAs). Five oral and maxillofacial radiologists individually assessed the radiographs. Area under the receiver operating characteristic curve (AUC), sensitivity, and specificity were calculated from the responses of the five examiners and compared between the devices tested using Student’s t test. Significance level was set at 5% (α = 0.05). The weighted Kappa index evaluated the intra- and inter-examiner agreements for caries lesions diagnosis. The use of a portable X-ray device did not influence on AUC, sensitivity and specificity metrics for the diagnosis of caries lesions (p > 0.05). The intra- and inter-examiner agreements for the caries lesions diagnosis ranged from substantial to almost perfect (0.646–0.859) and moderate to substantial (0.491–0.617), respectively. The diagnostic accuracy for detecting proximal caries lesions is not impaired when using a portable X-ray device.

## Introduction

The development of portable X-ray devices in the early 1990s emerged as an alternative to wall-mounted devices.^1,2^ Initially designed to aid dental care for soldiers in operations, these portable devices facilitated intraoral radiographic acquisitions in the military field.^1,2^ However, a previous study showed that over the past decades, portable X-ray devices have been implemented in clinical practice’s daily routines.^3^

Around 20,000 portable X-ray devices had already been sold worldwide by 2017.^4^ Portable X-ray devices, which may resemble a pistol or a camera, offer some vantages compared to wall-mounted devices, including reduced size, weight, and price.^2,4^ Although previous findings showed that the biological risks associated with the use of portable devices are no greater than those of wall-mounted device, their potential influence on diagnostic tasks has not been yet investigated.^5,6^

Caries remains as one of the most prevalent chronic diseases affecting people globally.^7^ Bitewing radiography is the recommended complementary examination for this diagnostic task, which may be challenging, especially when caries lesions are located in the proximal surfaces, preventing direct visual inspection.^8,9^ Moreover, for the radiographic detection of caries lesions, substantial demineralization (approximately 40%) must have affected the tooth, thereby increasing the complexity for diagnosing this task in early stages.^9^

Previous studies concluded that the use of different types of image receptors, such as analogue films, photostimulable phosphor (PSP) plates, and solid-state sensors do not influence the visualization of caries lesions.^10-12^ However, according to the consulted literature, the impact of a using portable X-ray device in the diagnostic accuracy for caries lesions had not been investigated yet. Therefore, the present study aimed to assess the influence of a portable X-ray device on the diagnosis of proximal caries lesions.

## Methods

This research study was conducted after approval of the local institutional Research Ethics Committee under the protocol number CAAE: 70610523.7.0000.5418 without any restriction and complied with the Helsinki Declaration.

### Sample selection and preparation

Posterior human teeth extracted for reasons unrelated to the present research and belonging to patients whose identification could not be determined (ensuring their anonymity and not requiring the need of applying an informed consent term) were selected. All these teeth presented white spots or other characteristics that could be suggestive of proximal caries lesion, such as color changes in enamel. Teeth with cavities extending to the dentin, restorations or anomalies were not included. Following the inclusion criteria, 40 teeth (20 premolars and 20 molars) composed the sample.

All 40 teeth underwent cleaning and subsequent disinfection with a 2% glutaraldehyde solution. Afterwards, each premolar was randomly paired with a molar and distributed among twenty silicone-phantoms. Each silicone-phantom included two non-test teeth (one premolar and one molar), simulating proximal contact, and totaling 20 silicone-phantoms. To simulate the opposing dental arch, one more silicone-phantom consisting of four posterior teeth was customized.

### Gold standard for caries lesions

To evaluate internal mineralization and confirm the presence of caries lesions in the mesial and distal surfaces, microcomputed tomography images of the forty teeth were acquired with the SkyScan 1174 device (Bruker Corp., Kontich, Belgium) set at 800 µA, 50 kVp, frame average of 1, 0.3˚ rotation step, 180˚ rotation, 15 µm pixel size, and 0.5-mm-thick aluminum filter.^13,14^

NRecon software v.1.6.8 (Bruker Corp., Konitch, Belgium) was employed to reconstruct the images. Beam-hardening correction of 35%, ring artifact correction of 5, and smoothing of 2 were applied to all images. These parameters mentioned were based on previous research studies.^14,15^ Two dentomaxillofacial radiologists (4 to 6 years of experience) evaluated, in consensus, the proximal surfaces of the teeth with the DataViewer software (Bruker Corp., Kontich, Belgium). As a result, it was found that 18 proximal surfaces exhibited caries lesions extending to the dentin-enamel junction, 35 surfaces showed lesions confined to the enamel, and 27 surfaces were sound.

### Radiographic image acquisition

Radiographs of the silicone-phantoms were obtained using an unused size-2 PSP plate of the VistaScan digital system (Dürr Dental, Bietigheim-Bissingen, Germany), and the DBSWIN Imaging software (Melville, New York, USA). The Eagle X-ray portable device (Alliage, São Paulo, Brazil) set at 2.5 mA, 60 kVp and an exposure time of 0.5 s was used. A fixed locator ring composed of acrylic was used to ensure standardized exposure geometry of the paralleling technique, including an object-PSP plate distance of 0.3 cm, focal spot-PSP plate distance of 40 cm, and vertical angulation of 90°. To mimic the X-ray attenuation caused by soft tissues, an acrylic block with a thickness of 2.5 cm was placed between the X-ray device and the silicone-phantom. In addition, to avoid impairing the quality of the radiographs caused by the operator’s movements, the portable device was positioned in a platform during all radiographic acquisitions. In all radiographic acquisitions the portable device was fully charged (Figure A).

**Figure:**
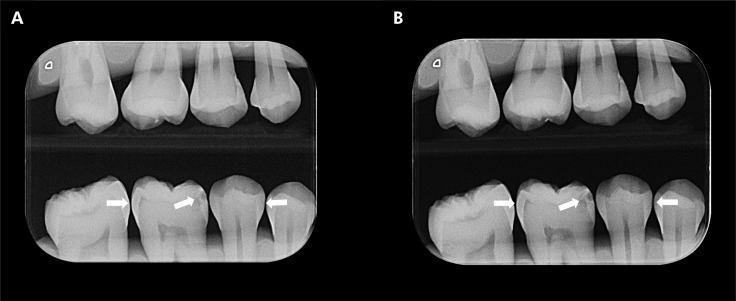
Bitewing radiograph of a silicone-phantom acquired with a portable X-ray device (A) and a wall-mounted device (B).

White arrows indicate proximal caries lesions.

Then, to comprehend the possible influence of the portable X-ray device on the diagnosis of caries lesions, new radiographs of the silicone-phantoms were acquired with a Focus X-ray wall-mounted device (Instrumentarium, Tuusula, Finland) set at 7 mA, 70 kVp, and exposure time of 0.16 s (Figure B). The same digital radiographic system and parameters mentioned for the X-ray portable device were applied.

The exposure times selected for the X-ray devices were determined after a pilot study, in which the authors subjectively evaluated different exposure times and their effects on the image quality. A duration of 0.16 s with a tube current of 7 mA was selected for the wall-mounted device for producing radiographs with satisfactory brightness and contrast. Additionally, a duration of 0.5 s with a tube current of 2.5 mA was selected for the portable device to maintain similar mAs level for both devices (1.25 mAs for the portable device and 1.12 mAs and for the wall-mounted device), avoiding potential biases related to the quantity of X-rays produced.

#### Image assessment

The resulting 40 radiographs (20 silicone-phantoms × 2 X-ray devices) were exported in 8-bit TIFF files and randomized. Five dentomaxillofacial radiologists, with more than five years of experience, evaluated the radiographs using JPEG view 1.0.35.1 software. The radiologists independently assessed the radiographs for proximal caries lesions, using a 5-point scale: 1 – absence, 2 – probable absence, 3 – uncertainty, 4 – probable presence, and 5 – presence. Each radiologist evaluated 160 proximal surfaces [20 phantoms × 2 devices × 4 surfaces (two from the premolar and two from the molar)], which were adequate to calculate the diagnostic metrics described in the statistical analysis. Prior to the image assessment, radiographs not included in the final sample were used to instruct and calibrate the examiners on the diagnosis of caries lesions.

The radiographic assessment occurred in a quiet and low-light environment. Examiners were instructed to evaluate 10 radiographs per day to prevent visual fatigue. Inter-examiner agreement was calculated based on these evaluations. Adjustments on brightness, contrast, and zoom were allowed, simulating common clinical practice. Twenty days following the end of the evaluation, 50% of the sample was randomized and reassessed to calculate intra-examiner agreement.

## Statistical analysis

SPSS 23.0 (SPSS Inc., Chicago, USA) software was used for statistical analyses. The significance level was set at 5% (α = 0.05). The area under the receiver-operating curve (AUC), sensitivity, and specificity were calculated for each examiner and device, and compared between portable and wall-mounted devices using the Student t test. The minimum difference between the averages of the groups, the average standard deviation, and the number of repetitions per group were used to calculate the power of the test, which was 75%. Intra- and inter-examiner agreements for the diagnosis of proximal caries lesions were evaluated by weighted Kappa index (0.00–0.20, slight; 0.21–0.40, fair; 0.41–0.60, moderate; 0.61–0.80, substantial; 0.81–1.00, almost perfect).^16^ The null hypothesis considered that the portable X-ray device would not be significantly different from the wall-mounted device on the diagnosis of proximal caries lesions.

## Results

Table 1 shows values of AUC, sensitivity, and specificity. The values of AUC (p = 0.635), sensitivity (p = 0.051), and specificity (p = 0.296) were not significantly changed by the use of a portable X-ray device when compared with the wall-mounted device. Moreover, based on the values of AUC, both X-ray devices resulted in acceptable discrimination (0.71–0.73) for the detection of proximal caries lesions.^17^

**Table 1:** Mean (standard deviation) values of diagnostic tests for proximal caries lesions using portable and wall-mounted X-ray devices.

Device	AUC	Sensitivity	Specificity
Portable	0.71 (0.03)	0.43 (0.09)	0.93 (0.05)
Wall-mounted	0.73 (0.07)	0.46 (0.08)	0.87 (0.10)
p-value*	0.635	0.051	0.296

AUC: area under the receiver operating characteristic curve. *according with Student t test.

Table 2 shows the intra- and inter-examiner agreements. While the intra-examiner agreements varied from substantial to almost perfect (0.646–0.859), the inter-examiner agreements varied from moderate to substantial (0.491–0.617).

**Table 2:** Intra- and inter-examiner agreements for the detection of proximal caries lesions.

Examiner	1	2	3	4	5
1	**0.859**	0.609	0.491	0.582	0.549
2		**0.739**	0.527	0.563	0.531
3			**0.646**	0.585	0.517
4				**0.727**	0.617
5					**0.731**

Bold values represent intra-examiner agreement.

## Discussion

Given that radiographic image quality depends on various technical parameters such as the type of X-ray device, X-ray beam geometry, and exposure time, this study aimed to compare the diagnostic accuracy of a portable X-ray device with a wall-mounted device for detecting proximal caries lesions using an *ex-vivo* setup.^1^ Previous investigation suggested that the image quality of the portable X-ray device might be inferior to that of a wall-mounted device.^18^ Consequently, it was hypothesized that the diagnostic performance of the portable X-ray device for detecting proximal caries lesions might be compromised. However, contrary to our hypothesis, the study’s null hypothesis was accepted. Both the portable and fixed-mounted X-ray devices exhibited comparable diagnostic performance, demonstrating acceptable discrimination in detecting the targeted diagnostic task.

The process of diagnosing caries lesions through radiographic images hinges on discerning density differences between dental hard tissues and affected areas (i.e., demineralized tissues).^19^ It is expected that images with improved contrast and radiographic density would aid in detecting such lesions more effectively, as these factors influence observers’ preferences.^14,20^ Notably, the lower image quality observed with portable X-ray device might be attributed to brightness, contrast, and image noise, all of which can impact the identification of caries lesions.^18^ Interestingly, there is a gap in the literature regarding a direct comparison of diagnostic performance between portable and fixed-mounted X-ray devices for dental diagnostic tasks to potentially confirm these hypotheses. To the best of our knowledge, only one previous study exclusively examined the diagnostic accuracy of a portable X-ray device for detecting proximal caries lesions using both analogue film and digital radiographs in an *ex-vivo* setting.^21^ Similar to our study, high AUC values were reported regardless of the image receptor used. However, the mentioned study did not compare the performance of the portable X-ray device with a wall-mounted device, as intended in the current study.

Although it may be deemed that the slight difference in kVp levels (i. e., 10 kVp) observed between the tested X-ray devices could influence the outcomes, several previous investigations had already demonstrated that variations in kVp do not affect the assessment of proximal caries lesions.^22-24^ For instance, previous studies indicated that a variation in kVp levels from 60 or 63 to 70 kVp does not impact the detection of proximal caries lesions or the measurement of periodontal bone levels.^24,25^ Similarly, Sogur et al. demonstrated that an increase from 50 kVp to 70 kVp did not affect the detection of proximal caries lesions in deciduous teeth.^23^ Additionally, another study showed that for different lesion detections, including proximal caries lesion and peri-implant bone defects, a variation in a higher range of kVp levels from 60 to 90 kVp also did not influence diagnostic performance.^22^

The portable X-ray devices operate on battery power. During clinical use, as the battery power decreases, there may be a gradual decline in the quality of the tube output, affecting both image quality and radiation safety.^1,10^ A previous study concluded that the tube voltage of various portable X-ray devices decreases as the battery charge level decreases, potentially impacting the quality of radiographic results.^10^ To prevent any potential bias in the outcomes assessed in the current investigation, all radiographs obtained with the portable X-ray device were obtained when the device was fully charged. However, future studies are encouraged to objectively and subjectively evaluate the influence of battery level on different diagnostic tasks.

In addition to monitoring battery levels, two other crucial factors were meticulously considered during image acquisition: ambient light exposure of PSP plates and the delay in PSP scanning after exposure. Both high ambient light exposure and delay in PSP scanning can negatively affect radiographic image quality, potentially introducing confounding variables into the present study. Therefore, for this investigation, all images were captured in a dimly lit environment, and the PSP plates were scanned immediately after exposure to X-rays.^26,27^

The present study is pioneering in conducting a diagnostic investigation on proximal caries lesions, comparing portable and wall-mounted X-ray devices. This aspect makes it challenging to directly compare our results with existing literature, yet it serves as a valuable reference for future studies. However, the literature offers limited studies that compare the overall image quality (i.e. not assessing a specific diagnostic task) between portable and wall-mounted X-ray devices. Pittayapat et al.^18^ assessed the overall subjective image quality of radiographs obtained from two portable X-ray devices and one wall-mounted X-ray device. In contrast to our study, one of the portable X-ray devices tested exhibited lower image quality than the wall-mounted device. Conversely, Nitschke et al.^28^ conducted a comparative study between a portable X-ray device and a wall-mounted one, considering various image quality parameters such as geometrical distortion and level of detail of different dental anatomical structures. Interestingly, similar to our study’s findings, both devices presented radiographic images with similar acceptable image quality.

The intra- and inter-examiner agreement ranged from moderate to almost perfect and from fair to moderate, respectively. In terms of intra-examiner agreement, our findings align with previous studies that examined similar diagnostic task concerning small non-cavitated proximal caries lesions.^14,15,29^ However, despite the inter-examiners’ agreement ranging from fair (0.49) to moderate (0.74), they surpassed those reported in the previous investigations.^14,15,29^This improvement can be attributed to the thorough calibration process conducted before the image assessment. It is essential to emphasize the reason for selecting teeth with incipient caries lesions. This approach aims to replicate a more realistic clinical scenario, as cavitated lesions are typically more easily identified by dentists and do not always require radiographic images. Including cavitated lesions in the present would possibly have reduced the diagnostic value of bitewing radiographs and not reflect the challenges in detecting early-stage caries lesions, preventing the authors from accurately assess the influence of using a portable X-ray device for this diagnostic task.

The present study has inherent limitations due to its *ex-vivo* setup, including the absence of clinical signs (e.g., pain and/or sensitivity) and potential patient movement. However, this study design is the only acceptable one that ensures the standardization of phantom positioning and allows for the acquisition of multiple radiographs from the same anatomical region solely for research purposes, which would not be feasible in a clinical setting. Moreover, the portable X-ray device was positioned on a platform during all radiographic acquisitions, preventing movements caused by the operator.

## Conclusion

The diagnostic accuracy for proximal caries lesions detection is not impaired when using a portable X-ray device.
